# Hypothalamic AMPK as a Mediator of Hormonal Regulation of Energy Balance

**DOI:** 10.3390/ijms19113552

**Published:** 2018-11-11

**Authors:** Baile Wang, Kenneth King-Yip Cheng

**Affiliations:** 1 State Key Laboratory of Pharmaceutical Biotechnology, The University of Hong Kong, Hong Kong, China; blwong@connect.hku.hk; 2 Department of Medicine, The University of Hong Kong, Hong Kong, China; 3 Department of Health Technology and Informatics, The Hong Kong Polytechnic University, Hong Kong, China

**Keywords:** hypothalamus, adenosine monophosphate-activated protein kinase, adipose tissue, food intake, adaptive thermogenesis, beiging

## Abstract

As a cellular energy sensor and regulator, adenosine monophosphate (AMP)-activated protein kinase (AMPK) plays a pivotal role in the regulation of energy homeostasis in both the central nervous system (CNS) and peripheral organs. Activation of hypothalamic AMPK maintains energy balance by inducing appetite to increase food intake and diminishing adaptive thermogenesis in adipose tissues to reduce energy expenditure in response to food deprivation. Numerous metabolic hormones, such as leptin, adiponectin, ghrelin and insulin, exert their energy regulatory effects through hypothalamic AMPK via integration with the neural circuits. Although activation of AMPK in peripheral tissues is able to promote fatty acid oxidation and insulin sensitivity, its chronic activation in the hypothalamus causes obesity by inducing hyperphagia in both humans and rodents. In this review, we discuss the role of hypothalamic AMPK in mediating hormonal regulation of feeding and adaptive thermogenesis, and summarize the diverse underlying mechanisms by which central AMPK maintains energy homeostasis.

## 1. The Hypothalamus and Energy Balance

The hypothalamus, a central integrator of the central nervous system (CNS), plays a critical role in the homeostatic regulation of appetite and energy expenditure by integrating hormonal, neuronal, and environmental signals [1]. It senses peripheral and central nutrients availability to modulate food intake and energy metabolism. Dysfunction of this highly-regulated system leads to energy imbalance, which initiates the development and progression of obesity and its related metabolic complications. There are different areas in the hypothalamus, which are believed to exert diverse functions in energy balance. Early in 1940, Hetherington and Ranson found that electrolytic lesions in the lateral hypothalamic area (LHA) cause inhibition of food intake, identifying LHA as a “feeding center” in the brain [2]. Subsequent studies showed that electrical stimulation of the LHA increases food intake [3], whereas lesions in the ventromedial hypothalamus (VMH) lead to the similar appetite-inducing effect [4]. Follow-up works demonstrated that not only lesions in the LHA and the VMH, but also disruption of other hypothalamic nuclei, including the arcuate nucleus (ARC), the dorsomedial hypothalamus (DMH), and the paraventricular nucleus (PVN), results in energy imbalance and obesity [5,6,7,8]. Among all of these regions, the ARC is critical in regulating feeding behavior and energy metabolism. The ARC is located near the median eminence (ME), which has abundant fenestrated capillaries that can lead to a ‘penetrable’ blood–brain barrier (BBB). The distinguished feature of the ME facilitates ARC neurons to sense hormonal and nutritional signals from the periphery [9], which is the reason why the ARC serves as the integration center of central and peripheral neural inputs.

The recent development of advanced techniques, including electrophysiology, optogenetics, and chemogenetics, enable us to identify distinct neuronal populations in the hypothalamus in rodents. The two best-studied neuronal populations in the ARC, which have opposite effects in appetite regulation, are the orexigenic Neuropeptide Y (NPY) and Agouti-related protein (AgRP) co-expressing neurons and the anorexigenic pro-opiomelanocortin (POMC) neurons. NPY/AgRP neurons are activated under a fasting condition, which drives hunger to promote food intake [10], whereas POMC neurons release alpha-melanocyte-stimulating hormone (α-MSH) to send satiety signals [11]. These two neuronal populations project to many second-order neurons in the PVN, VMH, DMH, and LHA [12,13,14]. The activity of these neurons is regulated by numerous neurotransmitters and/or hormones. For instance, the neurotransmitter serotonin exerts its anorexigenic effects by stimulating POMC neurons and suppressing NPY/AgRP neurons [15,16].

## 2. AMPK, an Energy Sensor and Regulator

AMP-activated protein kinase (AMPK), an evolutionarily conserved serine/threonine protein kinase, is a nutrient sensor that senses the ratio of AMP: adenosine triphosphate (ATP) or adenosine diphosphate (ADP): ATP to maintain energy balance in both peripheral tissues and the CNS. The heterotrimeric complex AMPK consists of three subunits, i.e., α catalytic subunit and β and γ regulatory subunits. Each subunit has several isoforms (α1, α2, β1, β2, γ1, γ2, γ3), suggesting 12 possibilities of heterotrimer combinations [17]. Some of these isoforms are tissue-specific and exert different functions under different physiological conditions [18,19]. For instance, heterotrimers containing the α1 isoform mainly exist in adipose tissues and the liver, whereas those containing α2 are predominantly expressed in skeletal muscles, the heart, and the brain [20,21]. The activity of AMPK can be regulated by both allosteric activation and phosphorylation at threonine 172 (Thr172) in the α-subunit. Specifically, allosteric activation is triggered by the increased intracellular AMP:ATP (or ADP:ATP) ratio, which facilitates the binding of AMP and/or ADP to the γ-subunit [22], while phosphorylation of AMPK is regulated by several upstream kinases, including liver kinase B1 (LKB1) [23,24], calcium-/calmodulin-dependent kinase kinase β (CaMKKβ) [25,26,27], TGFβ-activated kinase 1 (TAK1) [28,29], and the phosphatases including Mg^2+^-/Mn^2+^-dependent protein phosphatase 1E (PPM1E) [30], protein phosphatase 2A (PP2A), and protein phosphatase 2C (PP2C) [31]. The activated AMPK then shuts down ATP consumption and converts to ATP-producing pathways to stimulate carbohydrate and lipid metabolism by enhancing mitochondrial functions [21]. On the other hand, phosphorylation of AMPK at serine 485 (Ser485) in the α1 subunit or at serine 491 (Ser491) in the α2 subunit by protein kinase A (PKA) [32,33], autophosphorylation [32,34], or other protein kinases (such as Akt (also known as protein kinase B) [35,36] or the 70-kDa ribosomal protein S6 kinase (p70S6K) [37]) inhibits AMPK activity. The reduced AMPK activity in peripheral tissues, including liver, skeletal muscle, and adipose tissue causes glucose intolerance and lower exercise capacity, resulting in type 2 diabetes and obesity [21]. On the contrary, activation of AMPK by metabolic hormones, such as adiponectin and leptin, or a pharmacological compound, such as metformin, promotes insulin sensitivity and fatty acid oxidation in the peripheral tissues. Therefore, AMPK has been proposed as a promising drug target for obesity and type 2 diabetes [38,39].

## 3. Hypothalamic AMPK in the Regulation of Energy Balance

Apart from its crucial role in peripheral tissues, AMPK also plays a pivotal role in theCNS, especially in the hypothalamus. Activity of AMPK in the hypothalamus is induced by fasting but inhibited by feeding, hypothermia, and leptin, whereas high-fat diet (HFD) feeding blunts the leptin action and increases AMPK activity in the hypothalamus [40,41,42,43]. Specifically, AMPK activity is increased in AgRP-expressing neurons under fasting condition [44]. Hypothalamic AMPK modulates the functions of different neuronal populations (such as POMC and NPY/AgRP neurons), thereby controlling appetite and energy consumption to maintain energy homeostasis [45,46]. In addition, hypothalamic AMPK has been shown to control dietary selection, first- and second-phase insulin secretion, lipid metabolism, and hepatic gluconeogenesis, all of which are crucial for energy balance at the whole-body level [47,48,49,50]. Early studies revealed that pharmacological or adenovirus-mediated activation of AMPK in the medial hypothalamus significantly promotes food intake as a result of increased transcriptional levels of *NPY* and *AgRP* [51,52]. On the contrary, inhibition of AMPK by adenovirus-mediated overexpression of the dominant negative form of AMPK inhibits food intake [53]. Genetic-specific deletion of AMPKα2 in POMC neurons reduces energy expenditure and hence increases adiposity in mice, whereas deletion of this energy sensor in AgRP neurons prevents age-dependent obesity by promoting the anorexigenic effect of melanocortin [54]. Oh TS et al. recently demonstrated that AMPK regulates *NPY* and *POMC* transcription via autophagy in response to glucose deprivation in the mouse hypothalamic cell line [55]. A knockin mouse model with an activating mutation of AMPKγ2 (R302Q) gradually develops obesity due to elevated excitability of AgRP neurons and its associated hyperphagia [56]. Indeed, humans carrying this activating mutation have higher adiposity and dysregulated glucose balance [56]. Another protein-altering variant in AMPKγ1 has been recently shown to be associated with body mass index (BMI), which is identified by exome-targeted genotyping array [57]. Lentivirus-mediated overexpression of the constitutive active form of AMPK in corticotropin-releasing hormone (CRH) positive neurons in PVN leads to a food preference to a high carbohydrate diet over a HFD and obesity in mice [48]. In addition, AMPK activates the p21-activated kinase (PAK) signaling pathway in AgRP neurons, thereby mediating fasting-induced excitatory synaptic plasticity, neuronal activation, and feeding [44]. Apart from its direct action on POMC, NPY, and AgRP neurons, AMPK activity is also crucial to maintain excitatory synaptic input to AgRP neurons upon food deprivation [58]. In the following sections, we will discuss the key hormonal factors that positively or negatively regulate hypothalamic AMPK activity to control appetite and the underlying neuronal regulation.

## 4. Key Hormonal Factors That Regulate Food Intake via Hypothalamic AMPK

### 4.1. Leptin

Adipose tissue is an active and dynamic endocrine organ that secretes an array of hormones, bioactive peptides, and metabolites (collectively called adipokines), which control systemic energy, lipid and glucose homeostasis [59]. Leptin is the first identified adipokine that plays an indispensable role in controlling food intake and energy expenditure by mediating the crosstalk between adipose tissues and the hypothalamus [60]. The leptin receptor is abundantly expressed in POMC and NPY neurons in different regions of the hypothalamus [61,62,63]. Mutations in the *ob* gene (which encodes leptin) or the *leptin receptor* gene lead to severe obesity in humans and rodents mainly due to hyperphagia [64,65,66]. Leptin stimulates AMPK activation in skeletal muscle but reduces AMPK activity in the hypothalamus [38]. Noticeably, the inhibitory effect of leptin on AMPK activity is independent of the classic leptin signal transducer and activator of transcription 3 (STAT3) pathway [52]. The reduction of AMPK activity by leptin leads to an altered expression of neuropeptides, including NPY, AgRP, and α-MSH, in the ARC and the PVN [51,52]. Leptin selectively depolarizes POMC neurons and stimulates β-endorphin and α-MSH to inhibit AMPK activity [58,67,68]. AMPK also coordinates with other signaling networks, including mammalian target of rapamycin complex 1 (mTORC1) and phosphatidylinositol 3 kinase (PI3K), to fine-tune the hypothalamic actions of leptin [37,52,69]. For instance, PI3K-Akt-mTOR-p70S6K has been shown to phosphorylate AMPK at Ser485 and Ser491 in the hypothalamus upon leptin stimulation, which in turn reduces AMPK activity, leading to the inhibitory effect on food intake [37]. A more recent study also reports that leptin activates mTORC1 to repress AMPK activity via the PI3K-Akt axis [70]. Furthermore, the well-established downstream targets of AMPK in the peripheral tissues, such as acetyl-CoA carboxylase (ACC) and peroxisome proliferator-activated receptor gamma coactivator 1-alpha (PGC-1α), have been shown to mediate the hypothalamic function of AMPK [71,72]. Inhibition of AMPK by leptin increases the intracellular level of malonyl-CoA in the ARC and palmitoyl-CoA in the PVN through ACC [71]. Pharmacological blocking of the increase of these fatty acids attenuates leptin-induced suppression of food intake. A subsequent study demonstrated that inactivation of ACC by knocking in Serine 79 and Serine 212 with alanine in ACC impairs appetite in response to both fasting and cold in mice [73]. Genetic deletion of PGC-1α in AgRP neurons but not POMC neurons blunts the anorexigenic effect of leptin [72]. At the molecular level, knockdown of PGC-1α significantly reduces the mRNA level of *AgRP* in an AgRP-immortalized cell line under starvation but not fed state [72].

### 4.2. Adiponectin

Adiponectin is the most abundant adipokine that exerts multiple beneficial effects on the cardiometabolic system mainly via its insulin-sensitizing and anti-inflammatory properties [74,75]. In contrast to the increased level of leptin, the circulating level of adiponectin is reduced in humans with obesity and diabetes [76,77]. Adiponectin promotes glucose uptake and fatty acid oxidation in the skeletal muscle, suppresses glucose production in the liver, and induces vasorelaxation in the blood vessels [74,75]. These metabolic and vascular actions of adiponectin are largely mediated by AMPK [74,75]. Apart from its endocrine actions in the peripheral tissues, adiponectin also regulates feeding and energy expenditure via the hypothalamus [78]. Adiponectin can be detected in the cerebrospinal fluid (CSF) of mice after intravenous injection of recombinant full-length adiponectin, which promotes adaptive thermogenesis in brown adipose tissue (BAT) via the sympathetic nervous system (SNS)-uncoupling protein 1 (UCP1) axis [79]. Subsequent studies demonstrate that adiponectin is also detectable in human CSF, despite some studies having argued that adiponectin cannot pass through the blood–brain barrier [80,81,82,83]. In stark contrast to its abundant expression in circulation, only a trace amount of the trimeric and low-molecular-mass hexameric form (~0.1% of serum concentration), but no high-molecular-weight form, of adiponectin can be detected in CSF [80]. Importantly, the key signaling molecules (including the adiponectin receptors AdipoR1 and AdipoR2, the adaptor proteins containing an NH_2_-terminal Bin/Amphiphiphysin/Rvs domain, a central pleckstrin homology domain, and a COOH-terminal phosphotyrosine binding domain (APPL)1 and APPL2) mediating adiponectin actions in peripheral tissues can also be detected in different regions of the hypothalamus [84,85,86,87,88]. With regard to feeding regulation, two early studies demonstrated opposite effects of adiponectin on food intake via distinct mechanisms in the hypothalamus [89,90]. The first study by Kubota et al. demonstrated that intravenous injection of full-length adiponectin increases AMPK activity in the hypothalamus, which in turn promotes food intake and decreases energy expenditure under a refeeding condition [89]. These adiponectin actions are abolished by siRNA-mediated knockdown expression of AdipoR1 or adenovirus-mediated overexpression of dominant negative AMPK. Genetic abrogation of adiponectin has a similar effect on hypothalamic AMPK activity and appetite. On the contrary, the study by Coope A et al. showed that intracerebroventricular (i.c.v.) injection of adiponectin reduces food intake via AdipoR1 in a fasted state [90]. Such change is accompanied by activations of insulin (increased phosphorylation of insulin receptor substrate 1[IRS1], Akt, and forkhead box protein O1 (FOXO1)) and leptin (STAT3 phosphorylation) signaling as well as an increase of AdipoR1-APPL1 interaction. Consistent with Coope A et al.’s study, a recent study demonstrated that i.c.v. injection of adiponectin decreases body weight as a consequence of reduced food consumption and increased adaptive thermogenesis in the BAT, and such effect of adiponectin is diminished in rats with a nutritional imbalance during their neonatal period [91]. On the other hand, the peroxisome proliferator-activated receptor (PPAR)γ agonist pioglitazone, a well-established insulin-sensitizing drug, boosts food intake and reduces energy expenditure by inducing adiponectin production in adipocytes, which in turn increases and decreases mRNA expression of *NPY* and *POMC* in the hypothalamus, respectively, via the AdipoR1-AMPK-dependent pathway [92]. Surprisingly, patch-clamp electrophysiology experiments reveal that adiponectin specifically depolarizes POMC neurons and inhibits NPY neurons in a PI3K-dependent and AMPK-independent manner [93]. The discrepancy of adiponectin actions on food intake and hypothalamic neuronal activity may be due to the different nutritional states and concentrations of glucose used in the experiments. Indeed, two recent studies from Yada’s research group show that adiponectin exerts opposite effects on feeding and POMC neuron activity under low and high glucose concentration, despite the fact that the suppressive effect of adiponectin on NPY neurons is independent of glucose [94,95]. In addition to its direct action on hypothalamic AMPK, several studies report that adiponectin is able to modulate the actions of insulin and leptin on the hypothalamus, thereby controlling energy homeostasis [89,90,93,96].

Recently, Okada-Iwabu et al. discovered an orally active synthetic small molecule of adiponectin receptor agonist (namely AdipoRon) [97]. Treatment with AdipoRon not only improves metabolic health but also prolongs the lifespan in obese and diabetic mouse models [97]. Intraperitoneal injection of AdipoRon attenuates corticosterone-induced body weight gain, depression, and neuroinflammation in mice, indicating that AdipoRon can penetrate and target the CNS [98]. Since AdipoRon has been proposed for the treatment of type 2 diabetes, it is, therefore, interesting to investigate whether AdipoRon has any effect on hypothalamic function, as adiponectin, in the regulation of feeding and energy expenditure.

### 4.3. Ghrelin

The stomach-derived hormone ghrelin, released during fasting, is the first circulating factor that has been reported to stimulate appetite in humans [99]. The orexigenic action of ghrelin is mediated by NPY and AgRP peptides [100]. Central or peripheral administration of ghrelin upregulates hypothalamic AMPK activity in both the ARC and the VMH in rats via growth hormone secretagogue receptor [51,101,102,103]. AMPK activation by ghrelin can be controlled at the transcriptional level by the transcriptional factor ALL1-fused gene from chromosome 4 (AF4), its upstream kinase CaMKKβ, the Sirtuin 1 (SIRT1)-p53 pathway, or glucose availability [104,105,106,107]. Inhibition of AMPK activity abolishes the orexigenic action of ghrelin [101,108,109,110]. On the contrary, knockin of an activating mutation in AMPKγ2 potentiates the orexigenic action of ghrelin under a refeeding condition [56]. There are multiple downstream targets of hypothalamic AMPK to mediate the orexigenic effect of ghrelin. First, ghrelin increases the release of intracellular Ca^2+^ to activate the CaMKKβ pathway, and, thus, facilitates AMPK phosphorylation in NPY neurons in the ARC [58,111,112]. Second, ghrelin activates AMPK and increases cytosolic Ca^2+^ in NPY neurons in the ARC [112]. Third, ghrelin triggers a hypothalamic mitochondrial function via uncoupling protein 2 (UCP2), which antagonizes the reactive oxidative species (ROS) production, allowing AMPK-mediated fatty acid oxidation for the support of synaptic plasticity and neuronal activation of NPY neurons [108]. Fourth, López et al. report that ghrelin inhibits fatty acid synthesis via the AMPK-ACC-dependent pathway, leading to reduced production of malonyl-CoA (the product of ACC), which in turn promotes carnitine palmitoyltransferase I (CPT1) activity in the mitochondria [101]. A subsequent study indicated that the regulation of ghrelin on fatty acid metabolism only occurs in the VMH but not the ARC [102]. Lastly, like leptin, ghrelin modulates the activity of presynaptic neurons that activate NPY neurons in AMPK-dependent and positive feedback loop manners [58].

### 4.4. Insulin

Insulin is exclusively produced by pancreatic β cells, and secreted in response to different nutrient stimuli, including glucose, fatty acid, and amino acids, after a meal. Apart from its glucose lowering and lipogenic actions, insulin also acts as an anorexigenic hormone. Insulin-deficient animals are hyperphagia, whereas their voracious appetite could be rectified by central administration of insulin [113,114]. Brain insulin resistance, a status in which neurons fail to respond to a physiological concentration of insulin, causes a dysregulation of energy homeostasis and cognitive functions [115,116]. The role of central insulin signaling in maintaining energy balance could be verified, at least in part, using neuron-specific insulin receptor knockout (NIRKO) mice. NIRKO mice have an elevated plasma insulin level, increased food consumption, and are susceptible to diet-induced obesity without alterations in brain development or neuronal survival [117]. In addition, i.c.v. injection of insulin [118,119,120] or insulin analogues [121] reduces both body weight and food intake, while intrahypothalamic infusion of an anti-insulin antibody results in opposite effects [122]. Insulin exerts a broad suppressive effect on AMPKα2 activity in different regions of the hypothalamus, including the PVN, the ARC and the LHA [52]. Indeed, the suppressive action of insulin on AMPKα2 activity is comparable to that of leptin [52]. Streptozotocin (STZ)-induced β cell loss and subsequent insulin deficiency lead to activation of AMPK and increase expression of NPY in the hypothalamus, resulting in hyperphagia in rats [53]. Insulin treatment reverses STZ-induced AMPK activation in the hypothalamus. Pharmacological or molecular inhibition of AMPK in the hypothalamus reverses STZ-induced hyperphagia [53]. Moreover, the inhibitory effect of insulin on AMPK activity and food intake can be further potentiated by i.c.v. injection of the amino acid taurine [123], and the effect of insulin on AMPK activation depends on the extracellular glucose concentration [124]. On the other hand, the anorexigenic action and the inhibitory effect of insulin on hypothalamic AMPK are largely abolished by cold exposure [125]. Apart from its direct action, Han et al. found that hypoglycemia triggered by insulin increases AMPKα2 activity in the hypothalamus [126]. This phenomenon was remarkable in hypothalamic ARC, VMH, and PVN [126]. Interestingly, insulin has been shown to inhibit AMPK activity by inducing phosphorylation of AMPK at Ser485 and Ser491 in skeletal muscle, ischemic heart, and hepatoma HepG2 cells in an Akt-dependent manner; however, whether insulin exerts a similar effect on hypothalamic AMPK phosphorylations in response to feeding remains elusive [35,127].

### 4.5. Glucagon-Like Peptide-1 (GLP-1)

GLP-1 is not only an incretin hormone secreted by intestinal L cells [128], but also a neuropeptide produced by preproglucagon neurons in the nucleus of the solitary tract (NTS) in the brainstem, which projects to hypothalamic nuclei to regulate appetite [129,130]. Hypothalamic GLP-1 level is reduced under fasting condition, while central administration of GLP-1 inhibits food intake in fasted rats [131,132]. This anorectic effect of GLP-1 is mediated by its inhibitory effect on fasting-induced hypothalamic AMPK activation [132,133]. HFD or central administration of fructose has been shown to inhibit the anorectic action of GLP-1 [134,135]. In addition, expression of the *proglucagon* gene (which encodes GLP-1) in the brain is regulated by transcription factor 7 like 2 (TCF7L2), which is associated with the risk of diabetes [136]. Transgenic overexpression of the dominant negative form of TCF7L2 driven by the proglucagon promoter represses the expression of GLP-1 in the brain, leading to a defective repression of AMPK activity in response to feeding. The defect can be reversed by treatment with the cyclic adenosine monophosphate (cAMP)-promoting agent forskolin, indicating that GLP-1 mediates its anorectic effect via the AMPK-PKA-cAMP axis [136]. Of note, this signaling axis also mediates the anorectic effect of the GLP-1 receptor in the hindbrain [137]. Similar to GLP-1, targeted injection of the GLP-1 receptor agonist liraglutide or exendin-4 into the VMH inhibits food intake in humans and rodents [138,139]. The anorexic effect of exendin-4 can be reversed by pre-injection with the AMPK activator AICAR in the VMH [139]. Further analysis reveals that mTOR but not ACC acts as a downstream mediator of AMPK for the hypophagic effect of exendin-4 [139].

## 5. The Role of Hypothalamic AMPK in the Regulation of Energy Expenditure

Total energy expenditure consists of basal metabolism, physical activity, and adaptive thermogenesis. Among these three components, adaptive thermogenesis in response to cold temperature or dietary intake is predominantly controlled by the hypothalamus. Adaptive thermogenesis is mainly mediated by BAT, which dissipates heat via the mitochondrial protein UCP1 in brown adipocytes. In the past few years, great advances have been made to broaden our knowledge on the inducible thermogenic adipocytes (beige adipocytes) in subcutaneous white adipose tissue (sWAT). Under certain circumstances (such as cold exposure, β-adrenergic stimulation, intermittent fasting, or exercise), beige adipocytes could be induced within WAT, especially in sWAT [140,141,142,143,144]. This process is called white fat beiging or browning, and is largely regulated by the crosstalk between the hypothalamus, the SNS, and adipose tissues. Beiging of WAT not only enhances energy expenditure, but also improves glucose metabolism, insulin sensitivity, and hyperlipidemia to ameliorate obesity and its related cardiometabolic complications [145,146,147,148]. The activation of BAT and beiging of WAT is, at least in part, controlled by the hypothalamus-SNS axis [149]. As the interscapular brown adipocytes only exist in human infants [150], and the gene expression profiles of the inducible UCP-1 positive cells in human adults share high similarities with mouse beige adipocytes rather than classical brown adipocytes [151,152], it is possible that induction of beige adipocytes in humans could be a potential therapeutic target for the prevention of obesity and its related metabolic syndromes.

An early study showed that whole-body depletion of AMPKα2 leads to elevated sympathetic activity and increased catecholamine secretion [153], suggesting the potential role of AMPK in beiging via the SNS. Indeed, emerging evidence suggests that numerous hormonal factors regulate adipose tissue beiging and adaptive thermogenesis via inhibition of hypothalamic AMPK activity, which will be further discussed in the following sections.

### 5.1. Leptin

As mentioned above, leptin is known to inhibit AMPK activity in the hypothalamus, which is accompanied by enhanced whole-body energy expenditure [52,60]. Mice with deletion of protein tyrosine phosphatase 1B (PTP1B), an inhibitor of leptin signaling, have diminished activation of hypothalamic AMPKα2, accompanied by an upregulation of UCP1 expression and mitochondrial density in BAT [154]. Central administration of leptin increases sympathetic outflow to adipose tissues via AMPKα2 [155]. Further studies demonstrated that sensitizing the leptin signaling in POMC neurons by deletion of PTP1B increases energy expenditure and promotes the conversion of WAT into BAT [156,157], although whether the inhibition of AMPK contributes to these changes is unknown. In addition, sympathetic denervation abolishes the potentiating effect of PTP1B deletion on WAT beiging [157]. These data collectively indicate that leptin regulates adaptive thermogenesis in adipose tissues via the SNS.

### 5.2. Thyroid Hormones

Thyroid hormones, including triiodothyronine (T3) and thyroxine (T4), have been found to raise energy expenditure via their peripheral actions in BAT or central action in the hypothalamus [158,159]. Stereotaxic injection of T3 (an active form of thyroid hormone) into the VMH (where AMPK and thyroid hormone receptors are highly co-expressed) stimulates SNS activity and BAT thermogenesis by inactivating hypothalamic AMPK via the thyroid hormone receptors [158]. Subsequent studies indicate that administration of T3 in the VMH but not the ARC is able to induce beiging in sWAT and thermogenesis via inhibition of hypothalamic AMPK activity [160,161]. Inactivation of the lipogenic pathway in the VMH attenuates the central action of T3 on BAT thermogenesis [158]. Genetic deletion of AMPKα1 in steroidogenic factor 1 (SF1) neurons in the VMH mimics the central effect of T3 on BAT metabolism [162]. Ablation of UCP1 completely abolishes the thermogenic action of central T3 administration [161]. At the molecular level, T3 relives endoplasmic reticulum (ER) stress and ceramide level in the VMH via an AMPK-dependent pathway, which has been shown to promote beiging in WAT and reduce obesity [162]. Substitution therapy with Levothroxin, a synthetic form of T4, promotes the basal metabolic rate and BAT activity in human subjects with a condition of hypothyroid state [163], but it remains unknown whether this is mediated by central or peripheral action of AMPK.

### 5.3. BMP8B

Bone morphogenetic protein 8B (BMP8B), a member of transforming growth factor β, acts both centrally and peripherally to increase BAT thermogenesis in female rodents [164]. mRNA of *BMP8B* can be detected in the brain and its receptors, including ALK4, ALK5, and ALK7, which are expressed in the VMH and the LHA. BMP8B-deficient mice display impaired thermogenesis and reduced AMPK activity in the VMH [164,165]. Acute i.c.v. injection of BMP8B in the VMH rather than the LHA enhances sympathetic outflow to BAT but not to the kidney, which can be abolished by expression of the constitutively active form of AMPK or potentiated by expression of the dominant negative form of AMPK in the VMH, respectively [164,165]. In addition, central administration of BMP8B exerts its thermogenic effect in BAT via upregulating orexin, a key modulator of BAT thermogenesis, in the LHA via glutamatergic signaling [165].

### 5.4. GLP-1

Activation of GLP-1 receptors in the hypothalamus not only inhibits appetite but also regulates BAT function. Stimulation of GLP-1 receptors by their agonist liraglutide in the VMH triggers both BAT thermogenesis and WAT beiging in mice, which are mediated by AMPK inhibition [138]. Central administration of the GLP-1 receptor agonist increases sympathetic outflow to BAT, leading to increased ability of glucose and lipid clearance and thermogenesis in BAT [166]. Although injection of liraglutide in DMH has no effect on BAT thermogenesis, injection of native GLP-1 in DMH increases the core body temperature and thermogenic program in BAT [138,167]. However, whether the thermogenic actions of native GLP-1 and exendin-4 are also mediated by AMPK signaling remains unclear, which warrants further investigation. With regard to human studies, the effect of GLP-1 receptor agonists and GLP-1 on energy expenditure remains inconclusive [168].

### 5.5. Estradiol

Estrogens are known to play a key role in the regulation of energy balance. The central action of estradiol on BAT thermogenesis has been recently identified and linked to the AMPK pathway [169,170]. Estradiol binds to its receptor (estrogen receptor α) in the VMH to diminish AMPK activity and enhances BAT thermogenesis without affecting feeding behavior [169]. Similar to the action of thyroid hormone, estradiol is able to relieve endoplasmic reticulum (ER) stress and reduce ceramide synthesis in the VMH, which in turn promotes BAT thermogenesis.

## 6. Conclusions and Future Perspectives

The diverse mechanisms driven by the hormonal factors convey on the hypothalamic AMPK signaling axis, supporting a critical role of AMPK in controlling feeding behavior and energy expenditure to maintain whole-body energy homeostasis (Table 1). AMPK in the VMH is crucial for BAT thermogenesis and beiging of sWAT, whereas AMPK in the ARC regulates food intake. Inhibition of AMPK activity by estradiol and thyroid hormone protects the hypothalamus from lipotoxicity and ER stress, which are the central pathogenic pathways that contribute to insulin and leptin resistance in obesity [171]. A recent study pinpointed that AMPK in SF1 neurons in the VMH regulates BAT thermogenesis via the SNS [172]; however, whether other hypothalamic neuronal population(s) mediates the inhibitory effect of AMPK activation on BAT functions remains unclear.

Considering the vital roles of hypothalamic AMPK, drugs that specifically target central AMPK are worth developing to prevent obesity and its related metabolic syndromes. As the regulatory effects of AMPK are differential in the periphery and centrally, the best therapeutic strategy is to specifically target hypothalamic AMPK without altering its functions in peripheral tissues. In this respect, the use of nanoparticles or exosomes [173], optogenetic neuromodulations [58], or chimeric proteins (targeting peptides associated with effective molecules or steroid hormones) [174,175], drawing from the implementations in other diseases, might be innovative strategies to achieve specific modulation of hypothalamic AMPK activity. However, despite the high specificity, we cannot exclude the possibility that these strategies may also affect other neuronal populations near the target hypothalamic region, which would result in limited efficacy and undesired side effects [176]. Another important issue is how to address the long-term influence of the altered hypothalamic AMPK activity. As AMPK is a canonical regulator of glucose and lipid metabolism, whether the sustained inhibition of hypothalamic AMPK may lead to lipotoxicity or other deleterious effects in neurons still needs further investigation. Taken together, great endeavors are required to advance our understanding of neuronal and hormonal regulation of hypothalamic AMPK, and AMPK in the hypothalamus will be a fascinating therapeutic target if we can address all of the above concerns properly.

## Figures and Tables

**Table 1:** Actions of hormonal factors on hypothalamic AMPK activity, food intake, and energy expenditure.

Hormonal Factors	Hypothalamic AMPK Activity	Food Intake	Energy Expenditure
Adiponectin	↑	↑↓	↑↓
Ghrelin	↑	↑	-
Leptin	↓	↓	↑
Insulin	↓	↓	↑
GLP-1 and its analogues	↓	↓	↑
Thyroid hormones	↓	-	↑
BMP8B	↓	-	↑
Estradiol	↓	-	↑
